# Case Report: Long term multimodality treatment of canine hepatocellular carcinoma with serial monitoring of serum alpha-fetoprotein

**DOI:** 10.3389/fvets.2026.1942682

**Published:** 2026-10-06

**Authors:** Tae-Jung Dan, Cheol-Ho Yang, Yeon-Jin Kim, Min-Hee Kang, Mi-Ae Kang, Kwang-Sup Lee, Hee-Myung Park

**Affiliations:** 1 Department of Veterinary Internal Medicine, College of Veterinary Medicine, Konkuk University, Seoul, Republic of Korea; 2 Times Animal Medical Center, Suwon-si, Gyeonggi-do, Republic of Korea; 3 Department of Bio-animal Health, Jangan University, Hwaseong, Republic of Korea; 4 VET and GENE, Seongnam, Republic of Korea

**Keywords:** alpha-fetoprotein, canine hepatocellular carcinoma, immunotherapy, interleukin-15, olaparib

## Abstract

A 13-year-old spayed female Shih Tzu presented with a several-month history of progressively increasing liver enzyme activities concurrent with managed hyperadrenocorticism. Diagnostic imaging revealed a solitary hepatic mass originating from the caudate process of the caudate lobe adhering to the caudal vena cava. Hepatocellular carcinoma (HCC) was confirmed via histopathological examination of an ultrasound-guided tru-cut biopsy, accompanied by a markedly elevated serum alpha-fetoprotein (AFP) concentration. Molecular targeted therapy with olaparib was initiated since complete surgical resection was unachievable due to tumor adherence to the caudal vena cava and conventional chemotherapy has limited efficacy for canine HCC, based on genomic profiling. Following objective tumor progression and worsening mass effect after 2 months, cytoreductive surgery was performed. Postoperatively, olaparib and canine interleukin-15 (cIL-15) immunotherapy were instituted to control potential micrometastases. The patient achieved clinical and radiographic stability for a 10-month follow-up period, accompanied by a significant drop in serum AFP levels. However, following the discontinuation of cIL-15 and subsequent reduction of olaparib dosage because of suspected adverse effects, serum AFP levels rebounded progressively, and tumor recurrence was confirmed via ultrasonography. The patient died 20 months after the initial detection of the mass. This case describes the clinical course of canine HCC managed with a multimodal therapeutic approach and highlights the potential utility of serum AFP as an adjunctive biomarker for disease monitoring.

## Introduction

Primary hepatocellular carcinoma (HCC) accounts for approximately 1% of all canine neoplasms and over 50% of primary liver tumors in dogs (1, 2). It predominantly affects geriatric patients, with a mean age of 12 years, and approximately 80% of cases occur in dogs older than 10 years (1). Canine HCC presents in three morphological patterns: massive, nodular, and diffuse. The massive form comprises more than half of cases and typically appears as a solitary lesion in the left lateral, left medial, or caudate lobes, with reported metastatic rates ranging from 0 to 37%. In contrast, the nodular and diffuse forms each account for approximately 20% of cases and are associated with metastatic rates exceeding 90% (1, 3). Surgical lobectomy remains the treatment of choice for solitary massive HCC. When complete excision is achieved, median survival times can exceed 1,460 days (1). However, canine HCC is generally considered poorly responsive to conventional chemotherapy. In cases where complete surgical resection is not feasible and systemic chemotherapy is pursued, clinical outcomes are typically less favorable. One study reported a median survival time of 765 days in dogs without complete resection of massive HCC, compared with over 1,836 days in dogs that underwent complete excision (3).

Serum alpha-fetoprotein (AFP) has been investigated as a biomarker for HCC in both human and veterinary medicine. In humans, AFP is widely used for diagnosis and monitoring of treatment response, although its sensitivity and specificity are limited (4). In dogs, increased AFP concentrations have been reported in association with HCC; however, its clinical utility remains inconsistent, and its role in longitudinal disease monitoring has not been well established (5, 6). Therefore, further clinical data are needed to clarify the potential value of AFP as a biomarker in canine HCC.

Targeted therapy aims to interfere with specific molecular pathways involved in tumor development and progression. Although genomic profiling has facilitated the identification of actionable mutations in human oncology, its application in veterinary medicine remains limited, and the clinical relevance of many detected alterations is not fully defined (7–9). Immunotherapy, which enhances host immune responses against tumor cells, has emerged as an important therapeutic approach in human oncology and is increasingly being explored in veterinary medicine. Interleukin-15 (IL-15) is a γc family cytokine produced by multiple cell types that plays a central role in bridging innate and adaptive immunity. It promotes the proliferation and survival of T cells and natural killer (NK) cells and enhances cytotoxic immune responses (10–13). These properties support its potential application as an immunotherapeutic agent in cancer treatment.

The present case report describes the clinical course of a dog with HCC managed with a multimodal therapeutic approach, including cytoreductive surgery, targeted therapy based on tumor genomic profiling, and canine IL-15 (cIL-15) immunotherapy. In addition, serial measurement of serum AFP was performed to evaluate its potential role as a biomarker for disease monitoring.

## Case description

A 13-year-old spayed female Shih Tzu presented with a several-month history of progressive increases in serum liver enzyme activities. These abnormalities persisted despite management of previously diagnosed hyperadrenocorticism with trilostane (10 mg/dog, PO, BID; Vetoryl®; Dechra, Shrewsbury, UK) together with adjunctive treatment using ursodeoxycholic acid (7.5 mg/kg, PO, BID; Ursa®; Daewoong, Seoul, South Korea) and dietary management (Royal Canin® Hepatic; Royal Canin, Aimargues, France). The dog presented with mild polyuria, polydipsia, and polyphagia. The medical history included surgical excision of mammary gland tumors 1 year earlier, followed by postoperative cIL-15 therapy, with no evidence of recurrence during follow-up. Physical examination revealed abdominal distension and symmetrical truncal hyperpigmentation.

Complete blood count revealed lymphopenia and relative eosinopenia (Table 1, day 0). Serum chemistry revealed elevated alanine aminotransferase (423 U/L, reference range [RR] 17–78 U/L), alkaline phosphatase (2,649 U/L, RR 47–254 U/L), and γ-glutamyltransferase (259 U/L, RR 5–14 U/L).

**Table 1:** Complete blood counts and serum biochemistry data of a dog with HCC.

Parameters	D-147	D-84	D0*	D62	D118	D138	D201	D205	D226	D278	D348	D404	D439	D460	D488	D516	RR
RBC (×10^6^/μL)	–	–	6.63	–	7.16	–	–	5.73	5.53	7.01	6.69	7.06	6.5	6.92	5.76	5.94	5.50–8.87
HCT (%)	–	–	44.7	–	46.5	–	–	37.7	39.1	49.0	47.7	50.1	45.9	48.1	39.9	40.6	37.3–61.7
HGB (g/dL)	–	–	15.3	–	16.1	–	–	13.0	13.2	16.7	16.3	17.1	16.0	16.8	13.8	14.3	13.1–20.5
RET (×10^3^/μL)	–	–	26.5	–	76.6	–	–	44.7	21.6	69.4	58.2	66.4	56.6	49.1	48.4	36.0	10–110
PLT (×10^3^/μL)	–	–	372	–	**551**	–	–	385	282	353	343	352	348	405	398	454	148–484
WBC (×10^3^/μL)	–	–	7.55	–	8.64	–	–	11.14	7.71	7.02	6.53	9.20	8.43	7.51	11.42	10.00	5.05–16.76
NEU (×10^3^/μL)	–	–	5.90	–	6.87	–	–	9.08	6.22	5.78	5.32	7.65	6.84	6.32	9.18	8.38	2.95–11.64
LYM (×10^3^/μL)	–	–	**1.02**	–	1.09	–	–	1.06	**0.8**	**0.81**	**0.76**	**0.92**	**0.85**	**0.66**	1.45	**0.88**	1.05–5.10
MONO (×10^3^/μL)	–	–	0.51	–	0.54	–	–	0.66	0.62	0.37	0.39	0.59	0.66	0.50	0.76	0.7	0.16–1.12
ALT (U/L)	**210**	**277**	**423**	**422**	**627**	**620**	**689**	–	**204**	**260**	**227**	**212**	**246**	**288**	**332**	**447**	17–78
GGT (U/L)	**124**	**186**	**259**	**286**	**525**	**581**	**516**	–	**72**	**50**	**54**	**69**	**86**	**128**	**183**	**358**	5–14
ALP (U/L)	**1,953**	**2,696**	**2,649**	**2,743**	**Over 3,500**	**Over 3,500**	**Over 3,500**	–	**Over 3,500**	**Over 3,500**	**Over 3,500**	**Over 3,500**	**Over 3,500**	**Over 3,500**	**2,995**	**2,989**	47–254
BUN (mg/dL)	–	–	19.3	–	–	–	–	–	26.9	–	26.8	24.4	–	–	20.0	22.0	9.2–29.2
CREA (mg/dL)	–	–	0.43	–	–	–	–	–	0.4	–	0.34	0.38	–	–	–	0.32	0.4–1.4

*The initial detection of the hepatic mass; RR, reference range; RBC, red blood cells; CREA, creatinine. HCT, hematocrit; HGB; hemoglobin; RET, reticulocytes; PLT, platelets; WBC, white blood cells; NEU, neutrophils; LYM, lymphocytes; MONO, monocytes; ALT, alanine aminotransferase; GGT, γ-glutamyltransferase; ALP, alkaline phosphatase; BUN, blood urea nitrogen; CREA, creatinine.

Based on the patient’s medical history and pathological findings, suspicion of a primary hepatobiliary disorder was suspected. Accordingly, abdominal ultrasonography (US) was performed and revealed a solitary hepatic nodule (12.6 × 13.6 mm). As malignancy could not be excluded, computed tomography (CT) was performed 2 days later and revealed a solitary hepatic mass arising from the caudate process of the caudate lobe (Figures 1A,B). Although no evidence of regional lymph node or distant metastasis was identified, the mass was closely adherent to the caudal vena cava (CVC). Based on these findings, the primary differential diagnoses included malignant and benign primary hepatobiliary neoplasms. Owing to the marked adherence of the mass to the CVC, complete surgical resection was considered unlikely to be achievable. Therefore, cytoreductive surgery with histopathologic examination was recommended. However, the owner declined surgery because of financial constraints and elected to continue medical management of hyperadrenocorticism together with the existing supportive and dietary therapies. The dog subsequently developed intermittent anorexia and lethargy, and the hepatic mass was re-evaluated by ultrasonography 4 months after the initial diagnosis. Because the mass had increased substantially in size, indicating progressive growth, an ultrasound-guided Tru-Cut biopsy was performed. Histopathological examination of the Tru-cut biopsy specimen revealed a hepatocellular neoplasm composed of polygonal cells with abundant granular cytoplasm and round to oval nuclei. Mild to moderate anisocytosis and anisokaryosis were present, with occasional prominent nucleoli and multinucleated cells. Based on the overall histopathological features, the tumor was diagnosed as HCC. Serum alpha-fetoprotein (AFP) concentration was measured using a commercial assay (VET chroma™, ANIVET, Republic of Korea) and was elevated (280.97 ng/mL, RR < 70 ng/mL).

**Figure 1:**
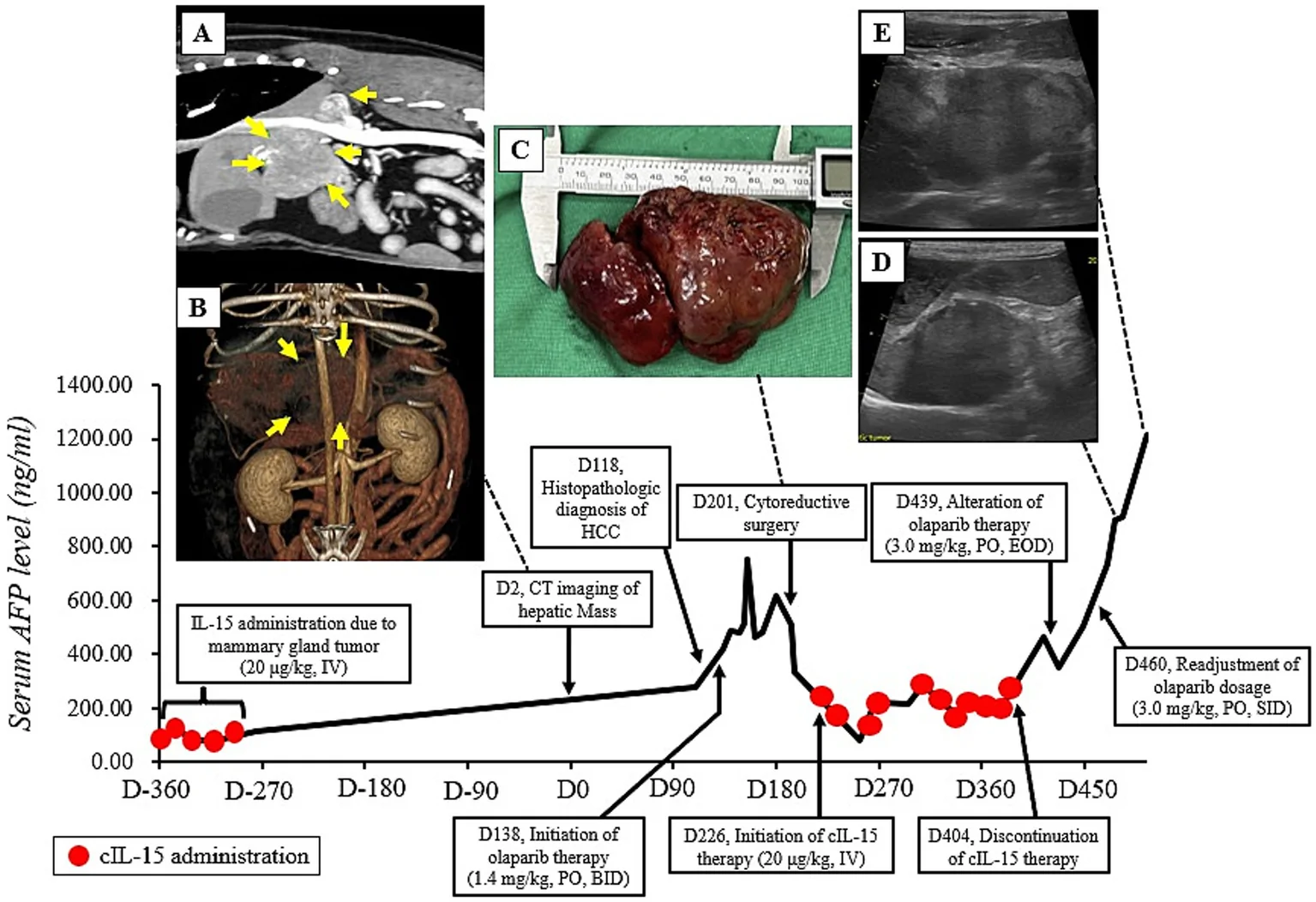
Serial serum alpha-fetoprotein (AFP) concentrations, treatment course, and imaging findings in a dog with hepatocellular carcinoma (HCC). The x-axis represents days from the initial detection of the hepatic mass (D0), and the y-axis represents serum AFP concentration (ng/mL). The line graph depicts serial changes in serum AFP concentration, with individual data points representing each measurement. Olaparib treatment was initiated on D138 at 1.4 mg/kg PO twice daily, cytoreductive surgery was performed on D201, and canine interleukin-15 (cIL-15) therapy was initiated on D226 at 20 μg/kg IV. Red dots indicate individual administration of cIL-15. **(A,B)** Show computed tomography (CT) images obtained on D2. **(A)** Standard CT image and **(B)** three-dimensional reconstruction of the hepatic mass. The mass is indicated by arrows, and peripheral contrast enhancement is evident. **(C)** Gross appearance of the resected hepatic mass. **(D,E)** Show abdominal ultrasonographic images demonstrating suspected recurrence of the hepatic mass. **(D)** On D488, the mass measured 2.76 × 3.63 cm, with serum AFP concentration of 901.04 ng/mL. **(E)** On D516, the mass measured 3.37 × 5.31 cm, with serum AFP concentration of 1,213.15 ng/mL.

Considering the limited efficacy of conventional cytotoxic chemotherapy for this tumor, a molecularly targeted therapeutic approach was pursued based on genomic profiling. The biopsy specimen was submitted for targeted cancer gene sequencing using a commercial panel targeting 120 cancer-related genes (SearchLight DNA assay; Vidium Animal Health, Long Beach, CA, USA), which identified an inactivating frameshift mutation (*p.Arg663*fs) in the *MEN1* gene. Although the clinical significance of this MEN1 alteration in canine HCC remains unknown, it was considered potentially relevant to tumor suppressor dysfunction. Based on this finding and evidence extrapolated from human oncology, olaparib (Lynparza®; AstraZeneca, Cambridge, UK) was initiated on an exploratory basis at 1.4 mg/kg PO twice daily, 5 months after the initial detection of the hepatic mass. Two months after initiation of olaparib treatment, follow-up CT revealed further tumor enlargement with increased lateral extension and mass effect, including greater compression of the CVC and pancreas, accompanied by a marked increase in serum AFP concentration (750.11 ng/mL). Given the risk of further disease progression with continued tumor enlargement and the absence of regional or distant metastasis, cytoreductive surgery was performed 6 days later (Figure 1C). The hepatic lobe containing the mass was resected by lobectomy. The tumor was carefully dissected from the CVC, and no gross residual tumor was evident at the completion of surgery. However, because the tumor was closely adherent to the CVC, a wide surgical margin could not be obtained at this site, and complete microscopic excision could not be confirmed.

Twenty-five days postoperatively, cIL-15 (Vaxleukin-15®; Vaxcell-Bio Therapeutics, Hwasun, Republic of Korea) was initiated at 20 μg/kg intravenously every 2 weeks to control potential micrometastases and prevent tumor recurrence. The olaparib regimen was adjusted to 3.0 mg/kg (PO, SID) owing to the owner’s difficulty in maintaining twice-daily administration, 8 months after initiation of olaparib treatment. At the time of this dose modification, the serum AFP concentration was 218.52 ng/mL, which remained lower than the preoperative value. The patient remained clinically stable throughout a 10-month follow-up period, with decreases in serum ALT and GGT activities and no ultrasonographic evidence of tumor recurrence. Serum AFP concentrations remained above the reference interval but showed no marked increase during this period (Figure 1). No clinically significant adverse effects were observed.

The cIL-15 regimen was discontinued approximately 9 months into the follow-up period at the owner’s request because of lethargy observed after administration. One month after discontinuation, serum AFP concentration increased from 208.49 ng/mL to 466.43 ng/mL, raising suspicion of tumor recurrence. However, at the owner’s request, the olaparib dosing regimen was subsequently reduced to 3.0 mg/kg PO every other day because of lethargy and hyporexia considered potentially treatment-related. Three weeks later, elevations in serum ALT (288 U/L) and GGT (128 U/L) activities (Table 1, day 460), and AFP (503.23 ng/mL) were observed. The olaparib dosage was readjusted to 3.0 mg/kg PO once daily. However, serum ALT and GGT activities and AFP concentrations progressively increased, with AFP reaching 1,213.15 ng/mL approximately 3 months later. Serial abdominal ultrasonography demonstrated a heterogeneous hepatic mass, measuring 2.76 × 3.63 cm at initial detection, with progressive enlargement on subsequent examinations. Although histopathologic confirmation was not obtained, the mass was considered suspicious for recurrent HCC (Figures 1D,E). The dog subsequently developed seizures and pulmonary edema and died at home 20 months after initial detection of the hepatic mass. The definitive cause of death could not be determined.

## Discussion

Standardized chemotherapy protocols for unresectable canine HCC have not been established, and available data remains limited. Previous studies have reported variable outcomes with cytotoxic chemotherapy and targeted agents, including gemcitabine, sorafenib, and toceranib (Table 2) (14–16). However, differences in tumor morphology, clinical stage, and treatment strategies across studies, along with small sample sizes, make direct comparison of therapeutic efficacy challenging, including in the context of the present case.

**Table 2:** Reported chemotherapy and targeted therapy protocols for unresectable or incompletely resected canine hepatocellular carcinoma.

Study (reference no.)	Treatment protocol applied	*n*	Tumor morphology and resectability	Surgical treatment	Clinical efficacy and survival outcomes	Adverse events (VCOG-CTCAE grade)
Elpiner et al. (14)	Gemcitabine 350–400 mg/m^2^ IV weekly for 5 weeks	18	Massive (*n* = 4; 2 incomplete, 2 unresectable) Nodular (*n* = 10; 9 incomplete, 1 unresectable) Diffuse (*n* = 4; all unresectable)	Incompletely excised (*n* = 11) Nonresectable (*n* = 7)	Overall: MST 983 d, PFI 971 d Unresectable subset: MST 197 d, PFI 150 d	Grade I-II Vomiting, anorexia, diarrhea, neutropenia and Grade III Neutropenia (*n* = 2)
Marconato et al. (15)	Sorafenib alone 5 mg/kg PO BID	5	Unresectable	No prior surgery (*n* = 3) Prior cytoreductive surgery (*n* = 2)	Individual TTP: 363, 1,579, 1,250, 228, 168 d Individual OS: 784, 1706 (alive), 1,398 (alive), 255, 361 d Response: CR (*n* = 1), PR (*n* = 3), SD (*n* = 1)	Grade II alopecia (*n* = 1), Grade I hyperpigmentation (*n* = 1), Grade I erythema/alopecia (*n* = 1) and Grade I diarrhea (*n* = 1)
	MC [Cyclophosphamide (10 mg/m^2^ q24h) Piroxicam (0.3 mg/kg q24h) Thalidomide (2 mg/kg q24h)]	6	Unresectable	No prior surgery (*n* = 2)	Individual TTP: 230, 205 d Individual OS: 327, 279 d Response: SD (*n* = 2)	None
	Sorafenib then MC crossover	2	Unresectable	No prior surgery (*n* = 5) Prior cytoreductive surgery (*n* = 1)	Median TTP: 27 d Median OS: 32 d Response: SD (*n* = 3), PD (*n* = 3)	Gastrointestinal; Grade I (*n* = 2), Grade II (*n* = 1), Grade I hemorrhagic cystitis (*n* = 1), Grade I renal toxicity (*n* = 1)
Heishima et al. (16)	Toceranib 2.0–3.0 mg/kg PO EOD (minimum 60 days)	6	Inoperable, massive (primary, metastatic, or recurrent lesion)	None	Response: PR (*n* = 3), SD (*n* = 1), PD (*n* = 2)	Grade I diarrhea, anorexia, neutropenia, lethargy and Grade II diarrhea (*n* = 1)

*n*, number of dogs; d, days; mo, months; MST, median survival time; PFI, progression-free interval; TTP, time to progression; OS, overall survival; CVC, caudal vena cava; CR, complete response; PR, partial response; SD, stable disease; MC, metronomic chemotherapy; PD, progressive disease.

Following cytoreductive surgery, the dog received olaparib and cIL-15 therapy. During more than 200 days of continued treatment, no hepatic mass was detected on ultrasonography, and serum AFP concentrations remained relatively stable. During the later follow-up period, cIL-15 was discontinued and the olaparib dosing regimen was modified by the owner. Serum AFP concentrations increased markedly (>1,000 ng/mL), and diagnostic imaging confirmed tumor recurrence with progressive enlargement.

Olaparib inhibits PARP-1, PARP-2, and PARP-3, thereby impairing PARP-mediated DNA repair and promoting synthetic lethality in tumor cells with impaired homologous recombination repair (HRR) (17, 18). The therapeutic efficacy of olaparib has been demonstrated in tumors harboring mutations in *BRCA1* or *BRCA2* (19, 20). In addition to *BRCA1/2* deficiency, deficiencies in several other proteins involved in homologous recombination repair and DNA damage signaling have been shown experimentally to increase sensitivity to PARP inhibition (21). In human HCC, PARP1 overexpression has been associated with poor clinical outcomes, and olaparib has been shown to suppress tumor growth (22).

In the present case, genomic analysis identified a frameshift alteration, *MEN1* p.Arg663fs. No reportable alterations were identified in key genes associated with homologous recombination repair (HRR) and DNA damage response, including *BRCA1/2*, *ATM*, *PALB2*, *CHEK2*, and *FANCA*, among those assessed by the 120-gene panel. Accordingly, *MEN1* p.Arg663fs was the only reportable genomic alteration identified among the 120 genes analyzed. *MEN1* encodes the tumor suppressor menin (23). Experimental studies have demonstrated that loss of *MEN1* function can impair homologous recombination-mediated DNA repair and increase sensitivity to the PARP inhibitor talazoparib in human pancreatic neuroendocrine tumor models (24). However, *MEN1* is not an established homologous recombination deficiency (HRD)-associated gene, and the presence of a *MEN1* alteration alone is insufficient to establish HRD and does not represent a validated predictive biomarker of PARP inhibitor sensitivity. Furthermore, the clinical relevance of *MEN1* alterations to PARP inhibitor responsiveness in canine HCC has not been established. Therefore, the use of olaparib in the present case was exploratory and was based primarily on evidence derived from human and preclinical studies.

Importantly, tumor progression occurred during preoperative olaparib monotherapy, and no therapeutic effect attributable to olaparib could be demonstrated. In the absence of reportable alterations in established HRR-associated genes or other evidence of HRD, the contribution of olaparib to subsequent disease control remains uncertain, and the possibility that it provided little or no therapeutic benefit cannot be excluded. The observed clinical course may instead have been influenced predominantly by cytoreductive surgery, with a possible contribution from cIL-15, as well as by the biological behavior of the tumor and other unmeasured factors. Further studies evaluating olaparib in canine HCC with confirmed HRD-associated alterations and in tumors lacking detectable canonical HRR-associated alterations are warranted to determine whether HRD status may serve as a predictive biomarker for response to PARP inhibition.

In addition to targeted therapy, cIL-15 was administered as an immunotherapeutic adjunct. IL-15 is a γc family cytokine that promotes the proliferation and activation of cytotoxic lymphocytes, including NK cells and CD8^+^ T cells, and enhances antitumor immune responses (10–13). In a study of healthy dogs, recombinant cIL-15 was well tolerated and induced expansion of anticancer effector cells without stimulating regulatory T cells (25). In addition, clinical studies in dogs with naturally occurring tumors, including lymphoma and mammary gland tumors, have demonstrated that cIL-15 administration in conjunction with conventional therapies was associated with improved tumor response rates, reductions in tumor-related biomarkers, and enhanced quality of life, with an acceptable safety profile (26, 27). These findings support the feasibility of cIL-15 as an adjunctive immunotherapeutic strategy in veterinary oncology, particularly for residual or micrometastatic disease. In the present case, cIL-15 was administered during postoperative period while the disease remained stable. However, its therapeutic contribution could not be directly demonstrated, and its role in disease control remains uncertain. The cIL-15 dose used in the present case (20 μg/kg IV) was selected based on a dosing regimen reported in a previous canine study (27). However, an optimal dosing regimen for cIL-15 in canine HCC has not been established and warrants further investigation.

AFP is a glycoprotein associated with fetal development, produced by the liver and yolk sac, and is typically present at low concentrations after birth due to suppression of gene expression (28, 29). In human HCC, AFP is elevated in approximately 60–80% of cases and is widely used for diagnosis, prognostication, and treatment monitoring (4, 29). In veterinary medicine, AFP has been reported to increase in dogs with HCC (6). A recent transcriptomic analysis demonstrated marked upregulation of *AFP* in canine HCC tissue relative to normal hepatic tissue (30). However, its diagnostic and monitoring utility remains inconsistent and is not yet well defined. In the present case, serum AFP concentrations exceeded the reference interval by more than tenfold prior to surgery, declined rapidly after cytoreductive surgery, and remained relatively low during the postoperative follow-up period before increasing markedly (1213.15 ng/mL) in parallel with tumor recurrence. Because the initial decrease in serum AFP occurred before initiation of cIL-15 therapy, it is most appropriately interpreted as primarily reflecting cytoreductive tumor removal, whereas the contribution of olaparib to this change remains uncertain. Previous reports have similarly documented a marked decrease in serum AFP after cytoreductive surgery (5, 6), suggesting that serial AFP concentrations may reflect changes in tumor burden over the clinical course, although their utility as a reliable biomarker in canine HCC remains to be established. Additionally, markedly elevated AFP concentrations have been reported to support suspicion of HCC, as concentrations in dogs with non-neoplastic liver disease and non-hepatic tumors are generally lower (6). However, given the limited data available in dogs and the potential overlap with other conditions, AFP should be interpreted cautiously and in conjunction with imaging and histopathological findings rather than as a sole biomarker.

This case has several limitations. As a single case report, it cannot establish the efficacy of olaparib or cIL-15 in canine HCC, and the individual contribution of each treatment remains uncertain. Furthermore, owner-initiated treatment modifications precluded evaluation under a consistent therapeutic protocol and limited interpretation of the clinical response to either therapy. Interpretation of olaparib efficacy was further limited by the lack of an established dosing regimen for canine HCC. Complete pathological staging was not possible because regional lymph nodes were not histopathologically examined, and the suspected recurrent hepatic mass was not histopathologically confirmed. Because necropsy was not performed, the final extent of disease and direct cause of death remained undetermined. Finally, the clinical utility of serial AFP measurements for monitoring disease progression and treatment response in dogs with HCC requires further validation.

In conclusion, this case describes the clinical course of a dog with unresectable HCC managed with a multimodal approach including cytoreductive surgery, targeted therapy, and immunotherapy. The observed temporal changes in serum AFP in relation to treatment and disease progression suggest that AFP may have potential utility as an adjunctive biomarker for monitoring disease course in canine HCC.

## Data Availability

The original contributions presented in the study are included in the article/supplementary material, further inquiries can be directed to the corresponding author.
